# Supplementary material for: Unraveling the developmental dynamic of visual exploration of social interactions in autism
Source: eLife. 2024 Jan 9;13:e85623. doi: 10.7554/eLife.85623 (PMC10876216; doi:10.7554/eLife.85623)
Supplement: MDAR checklist [file elife-85623-mdarchecklist1.pdf]

Westbrook Centre
Milton Road
Cambridge CB4 1YG, UK
P 01223 855340
W elifesciences.org
T @elife

Materials Design Analysis Reporting (MDAR) Checklist for Authors

The MDAR framework establishes a minimum set of requirements in transparent reporting mainly applicable to studies in the life sciences.

eLife asks authors to **provide detailed information within their article** to facilitate the interpretation and replication of their work. Authors can also upload supporting materials to comply with relevant reporting guidelines for health-related research (see EQUATOR Network), life science research (see the BioSharing Information Resource), or animal research (see the ARRIVE Guidelines and the STRANGE Framework; for details, see *eLife*'s Journal Policies). Where applicable, authors should refer to any relevant reporting standards materials in this form.

For all that apply, please note **where in the article** the information is provided. Please note that we also collect information about data availability and ethics in the submission form.

Materials:

Newly created materials	Indicate where provided: section/figure legend	N/A
The manuscript includes a dedicated "materials availability statement" providing transparent disclosure about availability of newly created materials including details on how materials can be accessed and describing any restrictions on access.		√

Antibodies	Indicate where provided: section/figure legend	N/A
For commercial reagents, provide supplier name, catalogue number and RRID, if available.		√

DNA and RNA sequences	Indicate where provided: section/figure legend	N/A
Short novel DNA or RNA including primers, probes: Sequences should be included or deposited in a public repository.		√

Cell materials	Indicate where provided: section/figure legend	N/A
Cell lines: Provide species information, strain. Provide accession number in repository OR supplier name, catalog number, clone number, OR RRID.		√
Primary cultures: Provide species, strain, sex of origin, genetic modification status.		√

Experimental animals	Indicate where provided: section/figure legend	N/A
Laboratory animals or Model organisms: Provide species, strain, sex, age, genetic modification status. Provide accession number in repository OR supplier name, catalog number, clone number, OR RRID.		✓
Animal observed in or captured from the field: Provide species, sex, and age where possible.		✓

Plants and microbes	Indicate where provided: section/figure legend	N/A
Plants: provide species and strain, ecotype and cultivar where relevant, unique accession number if available, and source (including location for collected wild specimens).		✓
Microbes: provide species and strain, unique accession number if available, and source.		✓

Human research participants	Indicate where provided: section/figure legend) or state if these demographics were not collected	N/A
If collected and within the bounds of privacy constraints report on age, sex, gender and ethnicity for all study participants.	The detailed demographics information of participants included in our study (age, gender and behavioral phenotype characteristics can be found in the *Methods and Materials section (Experimental Model and Subject Details).* The resumed version is available in the Table 1 of the manuscript.	

Design:

Study protocol	Indicate where provided: section/figure legend	N/A
If the study protocol has been pre-registered, provide DOI. For clinical trials, provide the trial registration number OR cite DOI.		✓

Laboratory protocol	Indicate where provided: section/figure legend	N/A
Provide DOI OR other citation details if detailed step-by-step protocols are available.		✓

Experimental study design (statistics details) *		
**For in vivo studies: State whether and how the following have been done**	**Indicate where provided: section/figure legend. If it could have been done, but was not, write "not done"**	**N/A**
Sample size determination	Although sample size had not specifically been estimated for our study, the final total number of participants included (N=217) is within a reasonable range when compared to other experiment in the field that include very young participants.<br><br>Detailed description of the sample is provided in the Methods and Materials, "Experimental Model and Subject Details "subsection. The sample included males with (166) and without (51) ASD for whom we acquired valid eye tracking data (we excluded data from participants who showed poor screen attendance, defined as binocular gaze detection on less than 65% of frames of the video).	
Randomisation	We did not perform randomization at the level of participant group attribution as the group definition are based on the DSM criteria as detailed in the Methods and Materials section. We perform randomization in some of our statistical analyses to prove the robustness of our analyses. Thus:<br>A) Randomization was conducted by permutation tests to test the significance of the difference in developmental trajectories between children with ASD and TD children, with details in ***Methods and Materials/*** *\Quantification and Statistical Analysis/ Quantifying the divergence in visual exploration &*	

	Results/ *Developmental patterns of visual exploration*. Figure 8, panel C depicts results of the permutation testing.<br><br>B) Additionally, permutations and bootstrapping were implements in all the analysis involving the Partial Least Squares (PLS) analysis. Details are provided in:<br><br>***Methods and Materials/*** *\Quantification and Statistical Analysis/ Multivariate association between gaze patterns and behavioral data*<br><br>***Results/*** *Less divergence in visual exploration is associated with better overall functioning in children with ASD. Figure 3*<br><br>***Results/*** *Developmental patterns of visual exploration. Figure 7& Appendix 4.*<br><br>C) Finally, we tested the stability of the referent gaze distribution depending on the sample size by performing bootstrap analyses. Details are provided in the Appendix 1.	
Blinding		✓
Inclusion/exclusion criteria	The details on the exclusion/inclusion criteria are available under Methods and Materials, “Experimental Model and Subject Details “subsection.	

**Sample definition and in-laboratory replication**	**Indicate where provided: section/figure legend**	**N/A**

State number of times the experiment was replicated in the laboratory.		√
Define whether data describe technical or biological replicates.		√

**Ethics**	**Indicate where provided: section/submission form**	**N/A**
Studies involving human participants: State details of authority granting ethics approval (IRB or equivalent committee(s), provide reference number for approval.	This information is available under Methods and Materials/Experimental Model and Subject Details/Cross-sectional sample and the Ethics statement in the submission form. The study protocol was approved by the Ethics Committee of the Faculty of Medicine of Geneva University, Switzerland (Swissethics, protocol 12-163/Psy 12-014, referral number PB_2016-01880). All families gave written informed consent to participate.	
Studies involving experimental animals: State details of authority granting ethics approval (IRB or equivalent committee(s), provide reference number for approval.		√
Studies involving specimen and field samples: State if relevant permits obtained, provide details of authority approving study; if none were required, explain why.		√

**Dual Use Research of Concern (DURC)**	**Indicate where provided: section/submission form**	**N/A**
If study is subject to dual use research of concern regulations, state the authority granting approval and reference number for the regulatory approval.		√

Analysis:

**Attrition**	**Indicate where provided: section/figure legend**	**N/A**
Describe whether exclusion criteria were pre-established. Report if sample or data points were omitted from analysis. If yes, report if this was due to attrition or intentional exclusion and provide justification.	Exclusion criteria were pre-established for data analysis. Such information was provided in the Methods and Materials/Quantification and Statistical Analyses:<br><br>We excluded data from participants who showed poor screen attendance, defined	

	as binocular gaze detection on less than 65% of video frames.	

Statistics	Indicate where provided: section/figure legend	N/A
Describe statistical tests used and justify choice of tests.	Statistical tests used are described in the Methods and Materials/ Quantification and Statistical Analysis and throughout the Results section:<br><br>1. Group comparison t-test :<br><br>Results/ Moment-by-moment divergence from the referent gazing patterns subsection and Figure 2.<br><br>Results/More ambient and less focal fixations in children with ASD compared to the TD group and Figure 4.<br><br>2. Multivariate pattern of association between the divergence of gaze behavior in autistic children and the referentTD grop and their clinical characteristics :<br>a. Crossectionnal sample : Results/Less divergence in visual exploration is associated with better overall functioning in chil-dren with ASD subsection and Figure 3.<br>b. Longitudinal sample : Results / More divergence in visual exploration is associated with unfolding autistic symptomatology a year later and Figure 7.<br>3. Multivariate pattern of association between the divergence of gaze behavior in autistic children and the referent TD grop and the characteristics of the movie : Results/ The association of movie content with divergence	

	in visual exploration in ASD group and Figure 5.<br><br>4. Group comparison using the nonparametric Wilcoxon test :<br><br>Results/ The relative contribution of the basic visual properties of the animated scene to gaze allocation in ASD and TD children, Figure 5 and Appendix 2. Effect sizes reported.<br><br>5. Permutation testing was done to test the significance of the between group difference in trajectories of gaze deployment:<br><br>Results/ Divergent developmental trajectories of visual exploration in children with ASD<br><br>We systematically reported exact p-values wherever possible alongside the summary statistics.	

**Data availability**	**Indicate where provided: section/submission form**	**N/A**
For newly created and reused datasets, the manuscript includes a data availability statement that provides details for access (or notes restrictions on access).	This information is provided in the *Data Availability* section in the submission form.	
When newly created datasets are publicly available, provide accession number in repository OR DOI and licensing details where available.		✓
If reused data is publicly available provide accession number in repository OR DOI, OR URL, OR citation.		✓

**Code availability**	**Indicate where provided: section/figure legend**	**N/A**

For any computer code/software/mathematical algorithms essential for replicating the main findings of the study, whether newly generated or re-used, the manuscript includes a data availability statement that provides details for access or notes restrictions.	This information is provided in the *Data Availability* section in the submission form.<br><br>The data and source code used to produce figures in the current paper will be uploaded to the GitHub repository shall the manuscript be accepted for publication. Shall the reviewers request access to the data and the source code for assessing this manuscript, we will be pleased to share them.	
Where newly generated code is publicly available, provide accession number in repository, OR DOI OR URL and licensing details where available. State any restrictions on code availability or accessibility.	This information is provided in the *Data Availability* section in the submission form.<br><br>The data and source code used to produce figures in the current paper will be uploaded to the GitHub repository shall the manuscript be accepted for publication. Shall the reviewers request access to the data and the source code for assessing this manuscript, we will be pleased to share them.	
If reused code is publicly available provide accession number in repository OR DOI OR URL, OR citation.	This information is provided Materials and Methods/ Multivariate association between gaze patterns and behavioral data:<br>The relation between behavioral phenotype and Proximity index was tested using the multivariate approach, Partial Least squares PLS-C Matlab-implemented source code publicly available on https://github.com/danizoeller/<br>Graph-Based Visual Saliency (MATLAB source code)<br><br>Materials and Methods/Proximity Index with regards to the visual properties of the animated scene:<br><br>To extract values of basic visual qualities of the scene, used the graph-based visual saliency (GBVS) version of the salience model (Matlab source code is publicly available https://github.com/Pinoshino/gbvs)	

Reporting:

The MDAR framework recommends adoption of discipline-specific guidelines, established and endorsed through community initiatives.

Adherence to community standards	Indicate where provided: section/figure legend	N/A
State if relevant guidelines (e.g., ICMJE, MIBBI, ARRIVE, STRANGE) have been followed, and whether a checklist (e.g., CONSORT, PRISMA, ARRIVE) is provided with the manuscript.		√

---

* We provide the following guidance regarding transparent reporting and statistics; we also refer authors to Ten common statistical mistakes to watch out for when writing or reviewing a manuscript.

Sample-size estimation

- You should state whether an appropriate sample size was computed when the study was being designed
- You should state the statistical method of sample size computation and any required assumptions
- If no explicit power analysis was used, you should describe how you decided what sample (replicate) size (number) to use

Replicates

- You should report how often each experiment was performed
- You should include a definition of biological versus technical replication
- The data obtained should be provided and sufficient information should be provided to indicate the number of independent biological and/or technical replicates
- If you encountered any outliers, you should describe how these were handled
- Criteria for exclusion/inclusion of data should be clearly stated
- High-throughput sequence data should be uploaded before submission, with a private link for reviewers provided (these are available from both GEO and ArrayExpress)

Statistical reporting

- Statistical analysis methods should be described and justified
- Raw data should be presented in figures whenever informative to do so (typically when N per group is less than 10)
- For each experiment, you should identify the statistical tests used, exact values of N, definitions of center, methods of multiple test correction, and dispersion and precision measures (e.g., mean, median, SD, SEM, confidence intervals; and, for the major substantive results, a measure of effect size (e.g., Pearson's r, Cohen's d)
- Report exact p-values wherever possible alongside the summary statistics and 95% confidence intervals. These should be reported for all key questions and not only when the p-value is less than 0.05.

Group allocation

- Indicate how samples were allocated into experimental groups (in the case of clinical studies, please specify allocation to treatment method); if randomization was used, please also state if restricted randomization was applied
- Indicate if masking was used during group allocation, data collection and/or data analysis
